# Core and Peripheral Criteria of Video Game Addiction in the Game Addiction Scale for Adolescents

**DOI:** 10.1089/cyber.2014.0509

**Published:** 2015-05-01

**Authors:** Geir Scott Brunborg, Daniel Hanss, Rune Aune Mentzoni, Ståle Pallesen

**Affiliations:** ^1^ Department of Psychosocial Science, University of Bergen, Bergen, Norway.; ^2^ Norwegian Institute for Alcohol and Drug Research, Oslo, Norway.

## Abstract

Assessment of video game addiction often involves measurement of peripheral criteria that indicate high engagement with games, and core criteria that indicate problematic use of games. A survey of the Norwegian population aged 16–74 years (*N*=10,081, response rate 43.6%) was carried out in 2013, which included the Gaming Addiction Scale for Adolescents (GAS). Confirmatory factor analysis showed that a two-factor structure, which separated peripheral criteria from core criteria, fitted the data better (CFI=0.963; RMSEA=0.058) compared to the original one-factor solution where all items are determined to load only on one factor (CFI=0.905, RMSEA=0.089). This was also found when we analyzed men aged ≤33 years, men aged >33 years, women aged ≤33 years, and women aged >33 years separately. This indicates that the GAS measures both engagement and problems related to video games. Multi-group measurement invariance testing showed that the factor structure was valid in all four groups (configural invariance) for the two-factor structure but not for the one-factor structure. A novel approach to categorization of problem gamers and addicted gamers where only the core criteria items are used (the CORE 4 approach) was compared to the approach where all items are included (the GAS 7 approach). The current results suggest that the CORE 4 approach might be more appropriate for classification of problem gamers and addicted gamers compared to the GAS 7 approach.

## Introduction

Video game addiction can be defined as “the persistent inability to control excessive gaming habits despite associated social or emotional problems.”^1^ One of the most frequently used instruments for measuring video game addiction is the Game Addiction Scale for Adolescents (GAS).^2^ The short version of the scale comprises seven items, each reflecting one of the following criteria: salience, tolerance, mood modification, withdrawal, relapse, conflict, and problems. The authors suggested that endorsement of all items, meaning that all seven criteria had occurred at least sometimes during the past 6 months, indicated video game addiction. This approach to measuring and defining video game addiction has been used frequently in the literature.^2–5^ However, it has also been argued that some of the seven criteria should be regarded as peripheral to video game addiction (i.e., salience, tolerance, and mood modification), whereas others more specifically relate to core criteria for addiction (i.e., withdrawal, relapse, conflict, and problems).^6–8^ The distinction is far from trivial, as the peripheral criteria seem to indicate high engagement with video games, whereas the core criteria seem to indicate problematic use of video games, or video game addiction. Addiction will usually involve high engagement. However, it is possible to be highly engaged without being addicted, and it is possible to be addicted without being highly engaged.^6^

One meta-analysis of prevalence and correlates of video game addiction concluded that studies focusing exclusively on the core criteria showed higher expected correlations with negative outcomes than studies relying on both peripheral and core criteria. In line with this, it was suggested that future studies should increase the focus on the latter type of criteria.^8^ One recent study used the GAS to categorize respondents as highly engaged gamers (those who endorsed all three peripheral criteria but no more than one of the core criteria), problem gamers (those who endorsed two or three of the core criteria), addicted gamers (those who endorsed all four core criteria), or non-problem/non-engaged gamers (all others).^6^ The study also assessed a number of subjective health complaints and found that problem gamers and video game addicts had greater risk of feeling low, feeling irritable or in a bad mood, feeling nervous, feeling tired and exhausted, and feeling afraid. The highly engaged gamers, on the other hand, did not have greater risk of experiencing these complaints compared to the non-problem/non-engaged group of respondents.

In sum, there are both theoretical and empirical indications that using all of the GAS's seven criteria might be a less than optimal approach to identifying video game addiction. The aims of the present study were therefore to investigate by means of confirmatory factor analysis (CFA) and multi-group invariance testing whether a two-dimensional structure of the GAS may be more suitable than the originally proposed one-dimensional structure and, consequently, whether using the four core criteria might be a better approach to identify video game addiction.

An implication of using only the core criteria is that the estimated prevalence of addiction will be slightly higher because endorsement of the peripheral criteria is not considered. Therefore, a secondary aim of this study was to compare the proportion of video gamers that can be classified as addicted to video games or problem gamblers when using either all seven criteria (both peripheral and core criteria) or, alternatively, only the four core criteria.

## Methods

Data were collected in 2013 as part of a survey of the Norwegian adult population. Postal invitations to take part in the study were sent to 24,000 individuals, aged 16–74 years old, randomly drawn from the Norwegian National Registry. A total of 10,081 individuals responded (response rate 43.6%, after removing those who were unable to take part or had invalid addresses). Only those participants who indicated that they had played video games during the previous 6 months (*n*=3,044) were asked to complete the GAS, and of these, 3,037 people completed the items. The mean age of this analytical sample was 35.0 years (*SD*=13.76 years), and 42% were female. Participants could answer a paper version or, alternatively, an online version of the questionnaire. One or two reminders were sent to those who did not reply.

### Measures

A Norwegian translation of the 7-item version of the GAS^2^ was used to measure video game addiction (Table 1). Responses were given on a 5-point scale ranging from 1=“never” to 5=“very often.” The original Dutch version of the GAS formed the basis for the Norwegian translation. Two bilingual (Dutch and Norwegian) researchers worked separately on the translation, and subsequently compared and revised the individual translations jointly to arrive at the final version used in the current study.

**Table 1.** One- and Two-Factor Solutions for the Game Addiction Scale

		*Two-factor solution*	
*How often during the last six months … (Hvor ofte i løpet av de siste seks månedene…)*	*One-factor solution*	*Factor 1 (peripheral criteria)*	*Factor 2 (core criteria)*
1. Did you think about playing a game all day long? (Tenkte du på spill hele dagen?)	×	×	
2. Did you spend increasing amounts of time on games? (Brukte du mer og mer tid på spill?)	×	×	
3. Did you play games to forget about real life? (Begynte du å spille for å slippe å tenke på andre ting?)	×	×	
4. Have others unsuccessfully tried to reduce your game use? (Spilte du videre, selv om andre ba deg stoppe?)	×		×
5. Have you felt bad when you were unable to play? (Følte du deg dårlig når du ikke kunne spille eller ikke fikk lov til å spille?)	×		×
6. Did you have fights with others over your time spent on games? (Havnet du i krangel med andre (f.eks. foreldre, venner, eller viktige andre) fordi du spilte for mye?)	×		×
7. Have you neglected other important activities to play games? (Lot du være å gjøre andre aktiviteter (f.eks. skole, jobb, lekser, idrett, hobbyer) for å spille?)	×		×

Norwegian translations of the items are given in parentheses.

### Analytical strategy

CFA was performed to test both the originally proposed one-dimensional structure^2^ and a two-dimensional structure of the GAS. In the two-dimensional structure, items 1–3 of the GAS were determined to load on one factor, named “peripheral criteria,” and items 4–7 were determined to load on the other factor, named “core criteria” (see Table 1). Mplus v5^9^ was used to perform CFA. No constraints were placed on the factor loadings. The factor variance was fixed to 1, and the factor mean was fixed to 0. The distribution of scores on the GAS items were skewed to the right. Thus. the assumption of multivariate normality associated with maximum likelihood estimation was violated. Therefore, the robust maximum likelihood (MLR) estimator was used.^10^ The proportion of missing values on the GAS items was small (seven respondents did not answer the GAS). Therefore, missing values were handled by listwise deletion. The Satorra–Bentler scaled chi square difference test^11^ was used to test whether the two models were significantly different from each other. Cutoff points for differences in the comparative fit index (CFI) and the root mean square error of approximation (RMSEA) suggested by Chen^12^ were used, where a difference in CFI of ≥0.010 and a difference in RMSEA of ≥0.015 are considered significant at the *p*<0.01 level.

The goodness of fit for the one-dimensional structure was compared to the goodness of fit for the two-dimensional structure for the total sample, and separately for men aged ≤33 years, men aged >33 years, women aged ≤33 years, and women aged >33 years.

Multi-group CFA was used to test the invariance of the measurement instrument for both the one-dimensional and two-dimensional structure. The invariance testing was conducted following the checklist by van de Schoot et al.^13^ and according to recommendations made by Vandenberg and Lance.^14^

The distributions of no-problem gamers, problem gamers, and addicted gamers were also compared using two approaches. Using the 7-item version, those who endorsed (indicated 3 or more on the 5-point response scale) all seven items were classified as addicted gamers, those who endorsed four to six items were classified as problem gamers, and those who endorsed fewer than four items were classified as no-problem gamers (“the GAS 7 approach”). Using a method called the “CORE 4 approach,” those who endorsed all four of the core criteria items (items 4–7) were classified as addicted gamers, those who endorsed two or three of the core criteria items were classified as problem gamers, and those who endorsed one or none of the core criteria items were classified as no-problem gamers.

## Results

### Comparing model fit

For the total sample, there was improvement in all goodness of fit indices from the one-factor model to the two-factor model (see Table 2). The goodness of fit statistics for the one-factor solution did not meet the cutoff values suggested in the literature,^15,16^ as the CFI was below the recommended value of 0.95 and the RMSEA was >0.08. The goodness of fit statistics for the two-factor model indicated a better fit with the data compared to the one-factor model. The CFI value was >0.95 and the RMSEA was <0.08, the cutoff value for good model fit.^10^ Overall, this indicates that the two-factor structure fitted the observed data better than the one-factor structure. Also, the Satorra–Bentler scaled chi square test revealed that the two models were statistically significantly different, χ^2^ (1)=160.47, *p*<0.01.

**Table 2.** Comparing Goodness of Fit Indexes for the One- and Two-Factor Solutions to the Gaming Addiction Scale

	χ^2^	Δχ^2^ (df)	CFI	ΔCFI	RMSEA	ΔRMSEA
*Total sample*						
One-factor solution	347.276 (14)^*^		0.905		0.089	
Two-factor solution	144.821 (13)^*^	195.511 (1)^*^	0.963	0.058^*^	0.058	−0.031^*^
*Men aged ≤33 years*						
One-factor solution	153.485 (14)^*^		0.886		0.110	
Two-factor solution	53.088 (13)^*^	135.08 (1)^*^	0.967	0.081^*^	0.061	−0.039^*^
*Men aged >33 years*						
One-factor solution	110.225 (14)^*^		0.900		0.103	
Two-factor solution	50.667 (13)^*^	49.05 (1)^*^	0.961	0.061^*^	0.067	−0.036^*^
*Women aged ≤33 years*						
One-factor solution	57.201 (14)^*^		0.939		0.057	
Two-factor solution	43.525 (13)^*^	10.84 (1)^*^	0.957	0.018^*^	0.050	−0.007
*Women aged >33 years*						
One-factor solution	71.161 (14)^*^		0.864		0.080	
Two-factor solution	46.049 (13)^*^	18.35 (1)^*^	0.921	0.057^*^	0.063	−0.017^*^

^*^ *p*<0.01.

CFI, confirmatory factor analysis; RMSEA, root mean square error of approximation.

This study also investigated whether the two-factor solution fitted the data better than the one-factor solution in four subgroups (men aged ≤33 years, men aged >33 years, women aged ≤33 years, and women aged >33 years). For all the subgroups, the goodness of fit was better for the two-factor solution compared to the one-factor solution. As shown in Table 2, Satorra–Bentler scaled chi square tests were statistically significant at the *p*<0.01 level for all subgroup comparisons. Differences in the CFI and the RMSEA were also significant, with the exception of only a small difference in the RMSEA for women aged ≤33 years.

### Testing measurement invariance for the one-factor solution

The first step of the invariance testing for the GAS with the one-factor solution was to test for configural invariance, that is, whether the one-factor solution was valid in each subgroup (see Table 2 for goodness of fit indexes). The goodness of fit was adequate for women aged ≤33 years (CFI=0.939; RMSEA=0.057). However, for the other three groups, the goodness of fit indexes indicated poor fit to the data. Hence, the one-factor solution failed the test of configural invariance. Unstandardized factor loadings (slopes) and intercepts for the one-factor solution across the four groups are shown in Table 3.

**Table 3.** Factor Loadings and Intercepts for the One-Factor Solution to the Gaming Addiction Scale

	*Men aged ≤33 years*	*Men aged >33 years*	*Women aged ≤33 years*	*Women aged >33 years*
*Factor loadings*				
Item 1	0.634	0.565	0.505	0.352
Item 2	0.683	0.667	0.599	0.634
Item 3	0.633	0.669	0.565	0.592
Item 4	0.735	0.551	0.385	0.345
Item 5	0.462	0.474	0.230	0.170
Item 6	0.538	0.370	0.224	0.134
Item 7	0.611	0.488	0.484	0.334
*Intercepts*				
Item 1	1.895	1.495	1.338	1.272
Item 2	2.151	1.775	1.529	1.625
Item 3	1.916	1.818	1.558	1.625
Item 4	1.710	1.415	1.263	1.223
Item 5	1.301	1.222	1.090	1.076
Item 6	1.360	1.154	1.106	1.060
Item 7	1.741	1.486	1.351	1.296

Because the one-factor solution was not configurally invariant, metric invariance, intercept only invariance, scalar invariance, and full uniqueness measurement invariance were not tested because configural invariance is required for subsequent tests of invariance to be meaningful.^14^

### Testing measurement invariance for the two-factor solution

The test of configural invariance for the two-factor solution showed that the goodness of fit was adequate for all four subgroups (Table 2). Three of the groups had CFI >0.95, and the fourth (women aged >33 years) had CFI >0.90, and all four groups had RMSEA <0.08. Hence, the two-factor solution CFA was valid for each group. The next step in the measurement invariance testing was to test for metric invariance, that is, whether the respondents in the different groups attribute the same meaning to the latent constructs. Following van de Schoot et al.,^13^ a model was tested where the factor loadings were held equal across groups, while the intercepts were allowed to differ across groups. Compared to the unconstrained model (CFI=0.963; RMSEA=0.058), the model testing for metric invariance (CFI=0.893; RMSEA=0.078) was significantly different (ΔCFI=−0.07; ΔRMSEA=0.02). Hence, the two-factor solution to the GAS items failed the test of metric invariance. Factor loadings and intercepts for the two-factor solution are shown in Table 4.

**Table 4.** Factor Loadings and Intercepts for the Two-Factor Solution to the Gaming Addiction Scale

	*Men aged ≤33 years*		*Men aged >33 years*		*Women aged ≤33 years*		*Women aged >33 years*	
	*Factor 1*	*Factor 2*	*Factor 1*	*Factor 2*	*Factor 1*	*Factor 2*	*Factor 1*	*Factor 2*
*Factor loadings*								
Item 1	0.761		0.643		0.534		0.402	
Item 2	0.838		0.782		0.641		0.721	
Item 3	0.633		0.669		0.552		0.552	
Item 4		0.771		0.556		0.404		0.391
Item 5		0.465		0.502		0.237		0.188
Item 6		0.578		0.393		0.239		0.161
Item 7		0.610		0.487		0.494		0.316
*Intercepts*								
Item 1	1.895		1.495		1.338		1.272	
Item 2	2.151		1.775		1.529		1.625	
Item 3	1.916		1.818		1.558		1.625	
Item 4		1.710		1.415		1.263		1.223
Item 5		1.301		1.222		1.090		1.076
Item 6		1.360		1.154		1.106		1.060
Item 7		1.741		1.486		1.350		1.296

Because the two-factor solution was not metrically invariant, intercept only invariance, scalar invariance, and full uniqueness measurement invariance were not tested, as metric invariance is required for subsequent tests to be meaningful.^14^

### Comparing classification of gamers: the GAS 7 versus the CORE 4 approach

The distribution of classifications (no-problem gamers, problem gamers, and addicted gamers) using all seven items (the GAS 7 approach) is cross-tabulated against the distribution of classification using only the core criteria (the CORE 4 approach) in Table 5. Using the GAS 7 approach, the proportions of gamers who were classified as no-problem gamers, problem gamers, and addicted gamers were 92.1%, 7.1%, and 0.7% respectively. Using the CORE 4 approach, the proportions of gamers classified as no-problem gamers, problem gamers, and addicted gamers were 92.3%, 6.6%, and 1.2% respectively. Thus, the proportion classified as no-problem gamers was highly similar using the two approaches, whereas the distribution of gamers classified as problem gamers or addicted gamers was slightly different.

**Table 5.** The Distribution of Problem Gaming Categories Using the GAS 7 Approach Cross-Tabulated Against the Distribution Using the CORE 4 Approach

	*GAS 7*			
*CORE 4*	*No-problem*	*Problem*	*Addicted*	*Sum*
No-problem	2,747 (90.5)	55 (1.8)	0 (0.0)	2,802 (92.3)
Problem	51 (1.7)	148 (4.9)	0 (0.0)	199 (6.6)
Addicted	0 (0.0)	14 (0.5)	22 (0.7)	36 (1.2)
Sum	2,798 (92.1)	217 (7.1)	22 (0.7)	3,037 (100.0)

The GAS 7 approach uses all seven items, whereas the CORE 4 approach uses only items 4–7 (core criteria). Numbers in parentheses are percentages of the total sum of gamers included in the sample (*n*=3,037).

GAS, Game Addiction Scale.

Of those who were classified as problem gamers using the GAS 7 approach, 27.1% (*n*=55) were classified as no-problem gamers using the CORE 4 approach. Conversely, of those who were classified as problem gamers by the CORE 4 approach, 27.6% (*n*=51) were classified as no-problem gamers by the GAS 7 approach. Of those who were classified as addicted using the CORE 4 approach, 38.9% (*n*=14) were classified as problem gamers by the GAS 7 approach.

## Discussion

Modeling the GAS items with a two-factor structure showed a better fit to the data compared to the one-factor structure suggested by Lemmens et al.^2^ Multi-group invariance testing showed that the one-factor solution to the GAS items failed the test of configural invariance, meaning that one cannot expect the one-factor solution to be valid in different subpopulations. The GAS does not measure the same latent factor in different subpopulations when the one-factor solution is applied. Therefore, it makes little sense to compare different sub-groups of the population (e.g., men vs. women; older vs. younger).^14^

For the two-factor solution, evidence was found of configural invariance across sub-groups, and more than adequate fit to the data in different subpopulations. This means that the individuals in the subgroups employed the same conceptual frame of reference when they responded to the questionnaire. This frame of reference involved a conceptual separation between two distinct dimensions. A reasonable interpretation is that the first dimension captures engagement with video games (see items 1–3 in Table 1), whereas the other dimension has to do with conflict and problems related to video games (see items 4–7 in Table 1). This corresponds well with what has been suggested in the literature.^6–8^ Researchers may use scores on the first dimension to determine level of engagement, and scores on the second dimension to determine level of video game addiction.

Using the two-factor solution, no evidence was found of metric invariance. Therefore, it cannot be expected that the regression slopes linking scores on the GAS items to the latent factors are equal across groups. This implies that while comparisons are viable between similar subpopulations (e.g., young men in different geographical areas), direct comparisons of different subpopulations (e.g., young men with older women) cannot me made without reservations. This may call for revising the GAS by adjusting or adding items.

Turning to categorization of groups of gamers, differences were found between using all seven GAS items (the GAS 7 approach) and using only the core addiction criteria (the CORE 4 approach). Using the CORE 4 approach, endorsement of all four core addiction criteria is needed to categorize someone as an addicted gamer, whereas with the GAS 7 approach, all three peripheral criteria must also be endorsed in order to be categorized as an addicted gamer. Therefore, a higher proportion of gamers were categorized as addicted when we used the CORE 4 approach instead of the GAS 7 approach. Whether the individuals identified as addicted using the CORE 4 approach are truly addicted to video games is a question that needs further investigation. In this regard, clinical comparison of gamers who are identified as addicted using the CORE 4 approach but not using the GAS 7 approach would be of particular interest. Also, studies investigating the relationship between addiction (comparing the CORE 4 approach and the GAS 7 approach) and correlates of addiction would be a welcome addition to the field in order to validate the suggested CORE 4 approach.

Several respondents changed categories from no-problem to problem gamers when the CORE 4 approach was used instead of the GAS 7 approach. The respondents who were classified as problem gamers using the GAS 7 approach, but as no-problem gamers using the CORE 4 approach endorsed all three peripheral items, but no more than one of the core criteria. If these items are not central to video game addiction, it may be the case that the classification of these respondents as problem gamers using the GAS 7 approach can be considered as false positives. Conversely, respondents classified as problem gamers using the CORE 4 approach but as no-problem gamers using the GAS 7 approach endorsed at least two of the core criteria, but no more than three of all seven items. Applying the same logic, these respondents may be considered false negatives when using the GAS 7 approach.

### Strengths and limitations

Some limitations should be addressed in future studies. A Norwegian version of the GAS was used. Replications of the present study in other languages are needed before more solid conclusions can be drawn. Another limitation is the relatively low response rate of the survey, meaning that the findings may not be representative of the whole population of gamers in Norway. Future studies may use other methods of data collection that have been associated with higher response rates, such as computer assisted telephone interviewing.^17^ It may also be questioned whether using four items is enough to classify gamers into different categories. Perhaps adding additional items that further tap the core criteria are needed for more robust categorization.

Still, some assets of the present study deserve mention. The nationally representative sample ensured high generalizability of the results. The large sample also provided strong statistical power and stable results. Use of Mplus for CFA enabled correction for skewed distribution of scores on the GAS items. Finally, the fact that clear differences were demonstrated between the one-factor and two-factor solutions across gender and age groups strengthens the validity of the conclusions drawn from the study.

### Implications

Separating the criteria peripheral to video game addiction from the core criteria is recommended. While the latent variable “engagement” can be measured by items that tap peripheral criteria, the latent variable “addiction” can be measured using items that tap the core addiction criteria. Because the measurement instrument failed to meet the requirements for metric invariance over subgroups, there is room for improvement to make the GAS better suited as a measurement instrument for video game engagement and addiction.

Another important implication is that the CORE 4 approach might be better than the GAS 7 for classifying video gamers as problem or addicted gamers. This is relevant both for future research and in clinical practice where an updated version of the GAS could be used as a screening tool for problems related to video game use.
